# Region-wise pattern of demographic, clinicopathological and treatment profile of thyroid cancers from 96 hospital-based cancer registries in India

**DOI:** 10.3332/ecancer.2025.1956

**Published:** 2025-08-01

**Authors:** Aleyamma Mathew, Preethi Sara George, Kondalli Lakshminarayana Sudarshan, Thilagavathi Ramamoorthy, Sreekumar Ananthakrishna, Elizabeth Mathew Iype, Anita Nath

**Affiliations:** 1 Regional Cancer Centre, Thiruvananthapuram 695011, India; 2 Indian Council of Medical Research, National Centre for Disease Informatics and Research, Bengaluru 562110, India

**Keywords:** clinicopathological, demographic, thyroid cancer, treatment, waiting-time distribution

## Abstract

**Introduction:**

Thyroid cancer (TC) is the most pervasive endocrine cancer worldwide. We examined the region-wise pattern of TC in India according to demographic, clinicopathological treatment and waiting-time distribution from diagnosis to treatment.

**Methods:**

TC cases in India from 96 hospital-based cancer registries (HBCRs) (North 28, East 8, West 7, South 40, Central 4 and North-East 9) reported for 2012-2019 were included. Among the 31,678 newly diagnosed cases, those treated only at the respective HBCR’s (*n* = 10,521) were included in the detailed analysis. Statistical significance by region was obtained using the chi-square test for categorical variables, the *t*-test for continuous variables and Marascuilo procedure to compare multiple proportions.

**Results:**

Among the 10,521 cases, 58% were from the South, the majority (68%) were females, with female-to-male ratio of 4.3:1, 3.8:1 and 3.5:1 in the north-east, central and southern regions, respectively, in the youngest (<35 years) age group (*p* < 0.001). The most familiar histological type was papillary carcinoma (69.0%). The female-to-male ratio for follicular carcinoma was 5:1 among <35 years, while the same for papillary carcinoma was 2.9:1. Distant metastasis at diagnosis was highest in the western region (19.0%), lowest in the South (13.4%). Radical intent-to-treat was highest in the South (92%) and lowest in the East (68.5%) (*p* < 0.001). Surgery alone or combined with other treatments was highest in the West (91.2%) compared to 48.9% in Central India. The waiting time from diagnosis to treatment was <7 days in 36% of cases from the North and the lowest proportion (19.0%) in both East and North-East regions (*p* < 0.001).

**Conclusion:**

We observed considerable heterogeneity in demographic, clinic-pathological, treatment and waiting time from diagnosis to treatment for TC across the regions in India; this kind of region-wise hospital-based analysis would help to improve national and local cancer care programmes.

## Introduction

Thyroid cancer (TC), a disease in which malignant cells form in the tissues of the thyroid gland, is the most pervasive endocrine cancer worldwide, accounting for 586,000 cases worldwide, ranking 9th place for incidence among all cancers. Women have a higher incidence of TC than men, especially in the reproductive age group. Globally, the TC incidence rate is approximately 10.0 per 100,000 among women and 3-fold higher than men [1]. A rise in the incidence (169%) and mortality (87%) of TCs was reported globally from 1990 to 2017 [2]. In India, it is estimated that 38,574 (29,037 women and 9,537 men) TC cases would be diagnosed in 2025. The highest age-standardised incidence rates among men (per 100,000) were 4.1 and 3.9 in Thiruvananthapuram and Kollam (South) districts. Among women, the highest rate (16.5) was in Papumpare (North-East) district, followed by 14.7 and 12.8 per 100,000 women in Thiruvananthapuram and Kollam districts [3].

TC includes mainly papillary carcinomas (PC), follicular (FC), medullary (MC) and anaplastic (AC) carcinomas. For clinical management, the disease is generally divided into two categories such as well-differentiated (papillary and follicular, highly treatable and curable) and poorly differentiated/anaplastic (less common, aggressive and metastasise early and poor prognosis). Medullary carcinoma, a neuroendocrine cancer, has an intermediate prognosis. Treatment management includes mainly thyroidectomy, I-131 ablation after surgery, external radiotherapy and targeted therapy according to the type and stage at diagnosis [4].

Some hospital-based studies in India reported that papillary carcinoma of the thyroid is the most common type [5, 6], most TC patients present with nodal metastases [7] and more than half of the patients with medullary carcinoma are found to have had distant metastases at the time of presentation [8]. However, studies examining the pattern of TC and its clinicopathological features across the regions in India are lacking. The present analysis aims to investigate the region-wise pattern of TC according to demographic, clinicopathological, treatment and waiting-time distribution from diagnosis to treatment using TC data from hospital-based cancer registries (HBCRs) in India [9].

## Materials and methods

This study includes TC (ICD-10:C73) [10] data from 96 HBCRs registered during 2012-2019 under the National Cancer Registry Programme (NCRP), Indian Council of Medical Research (ICMR), Government of India. HBCRs under the NCRP are usually located in specialised oncology centres/general or multi-speciality hospitals (public and private). HBCRs collect data on confirmed malignant cases (ICD-10: C00-C97) [10] from all departments and units where cancer is diagnosed or treated in the hospital. In order to eliminate duplicate case entries, only those cancer cases that were treated at the reporting institution were included in the analysis. This approach minimised inconsistencies due to prior diagnoses at external centers. Information on socio-demographic, diagnostic, clinical extent of disease (SEER 1977) and treatment details are collected on a standardised core form and entered into the software developed by the ICMR-National Centre for Disease Informatics and Research (NCDIR), Bengaluru. NCDIR assesses the data quality in dimensions such as validity, comparability, timeliness and completeness.

The 96 HBCRs' included in the study were divided according to their location into six regions of the country [North (*n* = 28), East (*n* = 8), West (*n* = 7), South (*n* = 40), Central (*n* = 4) and North-East (*n* = 9)]. In the present analysis, demographic characteristics included were age and gender, the diagnostic details were the date of diagnosis, the most valid method of diagnosis, topography and morphology using ICD-O-3 [11] and clinical extent of disease (CED). The CED was classified as localised (confined to the primary site), loco-regional (with spread to regional lymph nodes) and distant metastases (spread to different parts of the body) (SEER manual). Treatment-related factors were intention-to-treat (radical, palliative, pain relief and symptomatic treatment), type of cancer-directed treatment (CDT) (surgery, radiotherapy, chemotherapy, targeted therapy and combination of these treatments) given prior and at reporting institution (RI) separately and the date of initial cancer-directed treatment at RI.

A total of 31,678 newly diagnosed thyroid cancers were reported in these 96 HBCRs during the eight years, out of which 21,157 patients had received prior treatment at another health facility, while 10,521 had never received treatment earlier. A comparison of age and gender distribution of previously treated patients (*n* = 21,157) who attended the RI and the cases treated only at RI (*n* = 10,521) was also made.

## Statistical analysis

Mean and standard deviation (SD) were computed for quantitative data. The statistical significance of associations between the various qualitative parameters such as demographic, clinicopathological and treatment-related factors and the waiting-time distribution from diagnosis to treatment by region was evaluated through the chi-square (*χ*^2^) test. Continuous variables were analysed using independent *t*-tests for groups of two and one-way analysis of variance among groups of three or more. We used Marascuilo procedure [12] to compare the region-wise proportion of TC regarding gender, histology, the CED and intention to treat. *P* value ≤ 0.05 was considered statistically significant. All statistical analyses were performed using the SPSS 28.0 statistical package (SPSS, Inc., Chicago, IL, USA).

## Results

### Region-wise proportion of thyroid cancer compared to all cancers in the region

**Figure 1.**
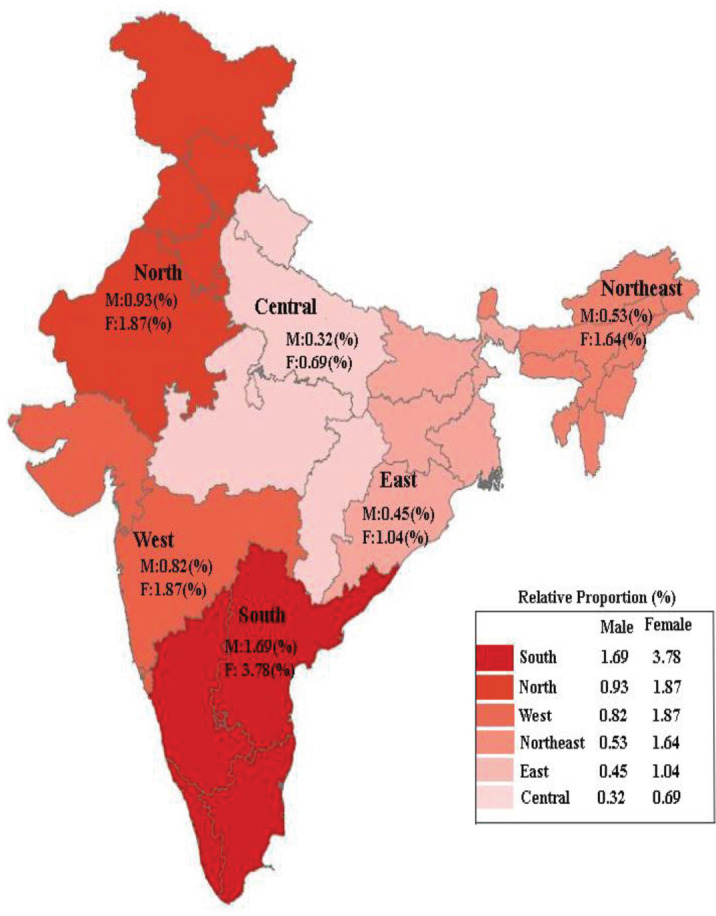
Region-wise proportion of thyroid cancer compared to all cancers in the region from HBCRs in India (2012–2019).

Of the 10,521 (males 3,340 and females 7,181) new TC cases, the highest relative proportion [compared to all cancers (ICD-10: C00-C97) in the respective region] was observed in the South (1.7% and 3.8%) and the lowest in the Central region (0.3% and 0.7%) for males and females, respectively. The region-wise proportional difference was statistically significant in both genders (*p*-value < 0.001 for both males and females) (Figure 1).

### Region-wise distribution according to gender and age

**Figure 2.**
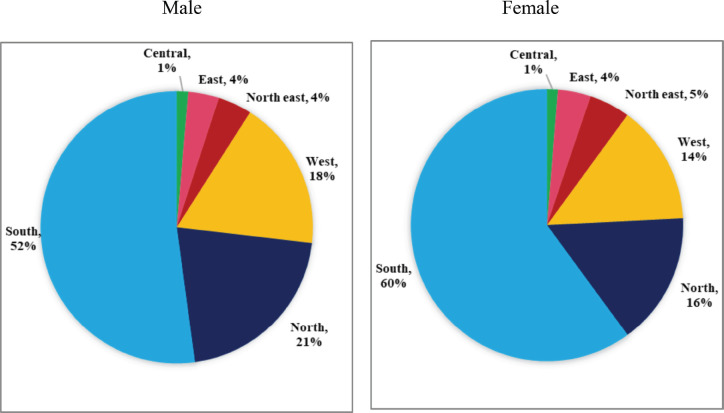
Region-wise distribution of thyroid cancer from HBCRs in India by gender.

The region-wise proportion of cases by gender is provided in Figure 2. Of the 10,521 TC cases, the highest proportion was reported from the South region (57.5%), followed by the North (17.4%), West (15.3%), North-East (4.8%), East (3.8%) and Central region (1.3%). The majority of cases were females (68.2%) with a female-to-male ratio of 2.1:1. However, the female-to-male ratio in <35 years of age was much higher than the overall value in the North-East (4.3 versus 2.3), Central (3.8 versus 2.0), South (3.5 versus 2.5) and East (3.3 versus 2.3) (Supplementary Table 1).

**Supplementary Figure 1.**
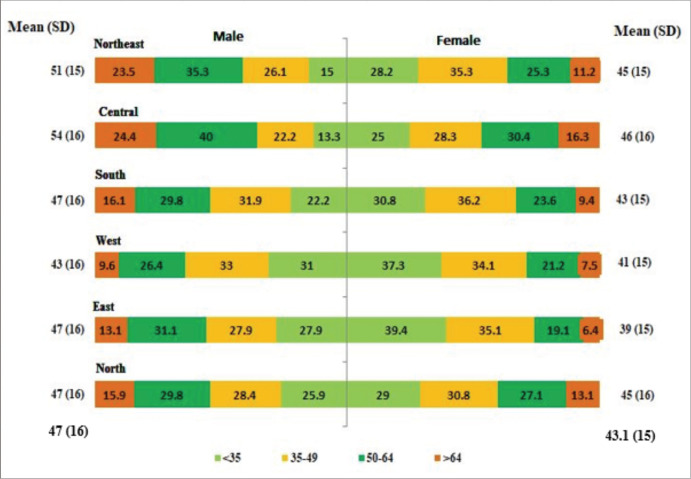
Region-wise thyroid cancer from HBCRs in India: mean age at diagnosis by gender *p-value for proportion: males < 0.001 and females < 0.001.

The mean age at diagnosis was 44.2 years (SD:15 years). Male patients were significantly older at diagnosis compared to female patients [mean age (SD): 47.0 (16) versus 43.1 (15) years for males and females, respectively, *p* < 0.001)] (Supplementary Figure 1).

**Supplementary Figure 2.**
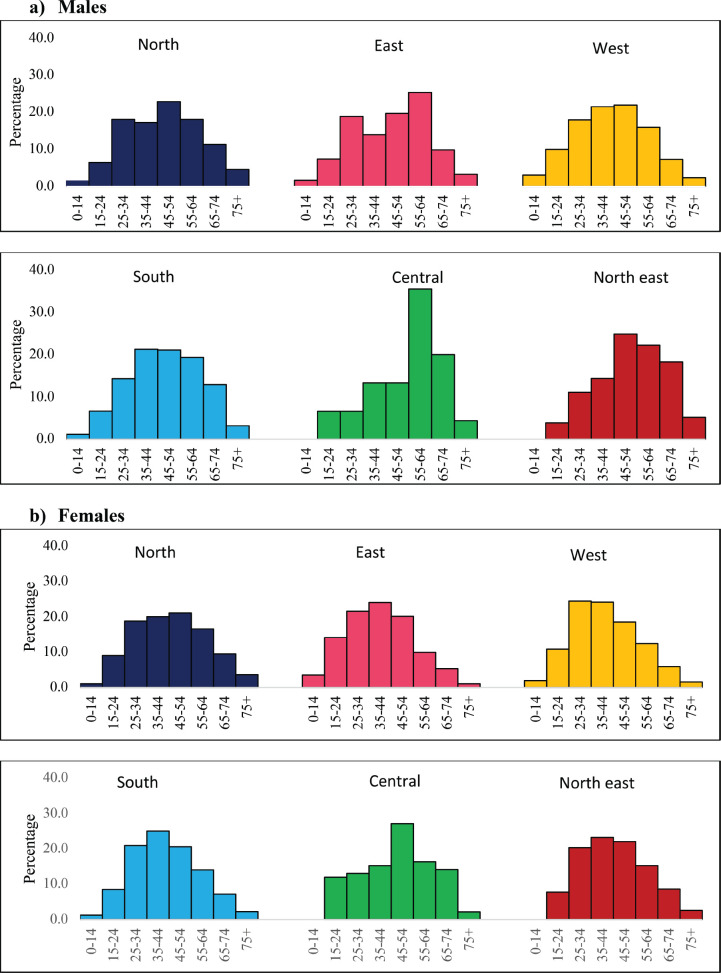
Region-wise thyroid cancer from HBCRs in India: age-distribution by gender a) Males b) Females.

Among females, 75% of cases were <50 years in the East, followed by 71.4% and 67% in the West and South, respectively (*p* < 0.001) (Supplementary Figure 2). Among males, 64% of cases were <50 years in the West, followed by 56% in the East and 54% in the South (*p* < 0.001). 4.1% of cases were in the age group <20 years, with 8.0% and 6.5% in the East and West, respectively. 68% of patients were Hindus, followed by 15% Muslims and 9.0% Christians.

### Histology

**Figure 3.**
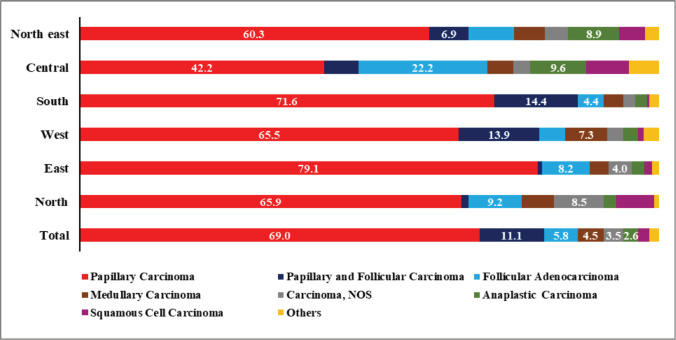
Region-wise thyroid cancers from HBCRs in India by histology. p-value for papillary carcinoma < 0.001 and follicular adenocarcinoma < 0.001 **denominator of proportion is microscopically diagnosed cases in the respective region.

The most common histological type was papillary (*n* = 7216, 69%), followed by follicular (*n* = 1158, 11.1%), medullary (*n* = 467, 4.5%), anaplastic (*n* = 268, 2.6%) and other rare types such as squamous cell carcinoma (SCC), insular carcinoma, sarcoma and so on (*n* = 375, 3.6%) (Figure 3). The female-to-male ratio for follicular cancer was much higher in the youngest age group (<35 years) (5.0:1) followed by 35–49 years (3.9:1) than the overall value (2.6:1). The same for papillary was slightly higher (2.9:1) in the youngest age group (<35 years) than the overall value (2.3:1) and the same for anaplastic was 3.9:1 in the age group 35–49 years, whereas the overall value was 1.6:1 (Supplementary Table 2).

### Clinical extent of disease

**Figure 4.**
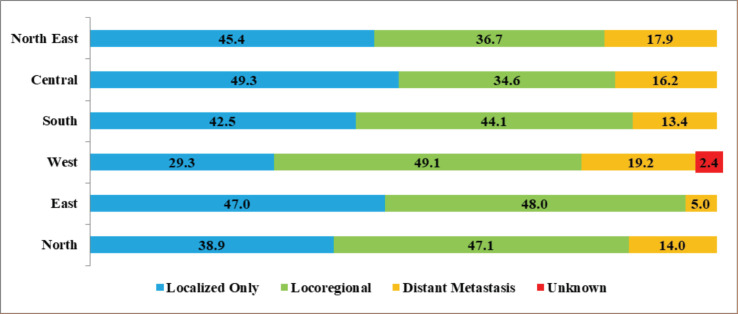
Region-wise thyroid cancers from HBCRs in India by clinical extent of disease * p-value for localised versus non-localised and region: <0.001; ** excluded unknown.

Thyroid cancers diagnosed in the stage with distant metastasis were higher in the West (19.2%), followed by North-East (17.9%), Central (16.2%), North (14.0%) and South (13.4%). The regional difference by CED (localised versus non-localised) was statistically significant (*p* < 0.001) (Figure 4). As expected, the proportion of anaplastic cancer in the localised stage was 9.8%, and the corresponding proportions in medullary, follicular and papillary were 30.1%., 41.0% and 43.3%, respectively. Compared to papillary (10.4%), a higher proportion of follicular cancers (31.2%) were diagnosed with distant metastases. A statistically significant difference was observed by CED and type of histology (*p*-value: 0.001) (Supplementary Table 2).

## Type of treatment

**Supplementary Figure 3.**
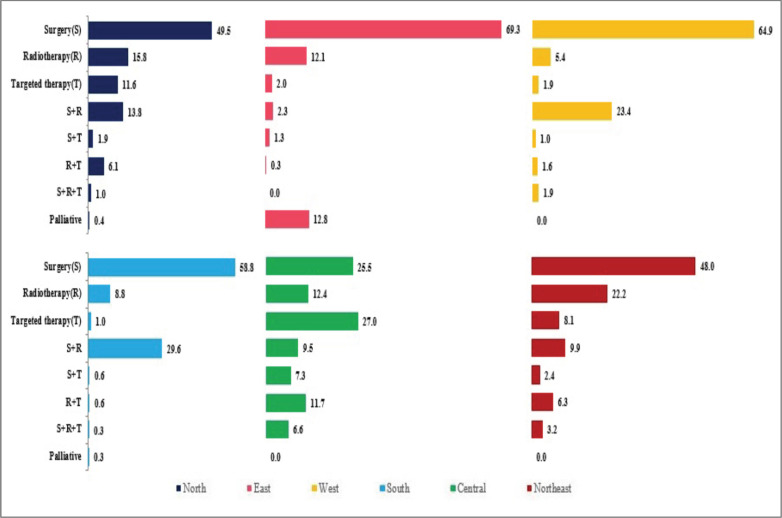
Region-wise thyroid cancer from HBCRs in India by type of treatment.

Of the 10,521 cases, nearly 80% had radical intention-to-treat, of which 8% had distant metastases (Table 1). The proportion of radical intention-to-treat was highest in the South (92.1%) followed by the West (89.4%) and North (82.6), and the same was lowest in the East (68.5%). However, the intention-to-treat in the East was unknown for 13% of cases. Surgery alone or with other combination of treatment was highest in the West (91.2%), followed by the South (89.3%) and East (72.9%), and the same was only in 48.9% of cases from the Central region (Supplementary Figure 3).

The proportion of patients who received CDT within 1 week after the diagnosis was highest in the North (36.2%), Central at 34.3% and South at 27.4%. However, the corresponding time was >2 months for 69.4% of patients in the West, 51.2% in the East, 47% in the North-east and 42% in the South (Table 2).

## Discussion

The present analysis provides a region-wise profile of TC in India, using 8-year data of nearly 100 HBCRs. We observed considerable heterogeneity in demographic, clinicopathological, treatment and waiting time distribution from diagnosis to treatment for TC across the regions. The majority of cases were females and from the South, the common histology was papillary. The female-to-male ratio for follicular was five-fold higher in women under 35 years, whereas the same for papillary was nearly 3-fold higher than men.

Population-based cancer registry figures provide regional differences more accurately; however, the present analysis, based on HBCR data provided a starting point for determining the regional distribution of thyroid cancer in India as we included thyroid cancer cases treated only at the respective HBCR’s and hence no duplicate cases are included in the analysis.

Among the total 10,521 cases, 58% were from Kerala. Population-based studies also recorded a high burden of TC in Kerala, South India. The higher figures could be attributed to increased awareness and easy access to ultrasonography [13–15]. Trends in thyroid cancer mortality, case fatality ratio and the proportion of micro-invasive carcinomas and early-stage cancers need to be assessed before concluding it as over-diagnosis. Also, specific studies are needed to identify whether the diagnosis is incidental due to the use of ultrasound in evaluating other diseases.

In the present analysis, we observed that nearly 70% of the cases were females. A higher female-to-male ratio was observed in the youngest age group (<35 years) in the North-East (4.3:1), Central (3.8:1) and South (3.5:1) as well as in 35–49 years. A meta-analysis supported an association attributed to changes in female hormones during the menstrual cycle and pregnancy with the risk of TC, which explains female preponderance [16].

Some hospital-based studies have reported that the incidence of TC is increasing in India, particularly among the younger population (<45 years) [8]. In the present analysis, we observed that the relative proportion of TC was approximately 52% in those under 45 years. As expected, papillary carcinoma was the most familiar histological type, followed by follicular, which agrees with study findings from other countries [17, 18]. Follicular cancer occurs at a younger age than papillary, particularly among women. In the present study, we observed that FC was five-fold higher in <35 years, whereas the same for PC was nearly 3-fold higher in women than men. Studies have reported that an aggressive type of TC is ACs, which have similar incidence rates in men and women and occur at older ages. In the present series, AC had a slightly higher proportion in females (female to male ratio was 1.6:1), with a mean age at diagnosis somewhat higher than the PC, and FCs and MCs were more or less equally distributed in different ages in both genders in all the regions. Even though it is a rare pathological type, studies have reported that MCs occur more frequently in males than females [19].

The present analysis noted that the proportion of cases detected in the localised stage was lower (42.5%) in the South compared with 49.3%, 47.0% and 45.4% in the Central, East and North-East regions, respectively. At diagnosis, distant metastasis was seen in 19% of cases in the West, and the same was the lowest (13.4%) in the South. Studies have reported that papillary thyroid cancer may spread to nearby lymph nodes in the neck with a good prognosis. Follicular cancer does not usually spread to nearby lymph nodes, but they are more likely than PC to spread to other organs, like the lungs or the bones. Medullary cancers are more likely to spread to lymph nodes and other organs; an anaplastic carcinoma is an aggressive form that quickly spreads to different parts of the neck and body [20].

There was a wide variation in the intention-to-treat and the type of treatment in the present series. Radical intent was highest in the South (92%) and the lowest (68.5%) in the East. Surgery alone or combined with other treatments was highest in the West (91.2%), whereas only 48.9% in Central. Early diagnosis and appropriate treatment can improve prognosis and reduce mortality. The increasing treatment options for thyroid cancer patients in the US, including therapies approved by the US Food and Drug Administration, have kept the mortality rate from this malignancy low despite the increase in its incidence [21].

In the present series, it was observed that the intent-to-treat was palliative/symptomatic in 2% of cases in the localised and 8% in the loco-regional stages. Furthermore, 16% of patients in the localised stage received only radioiodine therapy and/or other systemic therapy. In a recent comprehensive study involving 11,570 patients, it was observed that aggressive PC accounted for 1.5% of cases. The study found that tumours equal to or smaller than 1 cm demonstrated a significantly higher incidence of extrathyroidal extension and neck node metastasis. As a result, the study suggests that surgeons should take into consideration the presence of aggressive subtypes as crucial factors when determining the extent of surgery for papillary carcinomas smaller than 1 cm [22].

Regarding the waiting time from diagnosis to treatment, it was <7 days in 36% of cases from North and the lowest proportion in East and North-East (19%). However, the corresponding time was >2 months in 69.4% in the West, 51.2% in the East, 47% in the North-east and 42% in the South. Studies have reported that the impact of extended patient waiting times, from initial diagnostic imaging and FNAC to thyroid lobectomy, subsequent completion surgery and onward to radioiodine therapy, is extensive, resulting in substantial psychological morbidity [23].

The main strength of this analysis is that it reports the demographic, clinicopathological and treatment status of thyroid cancer data from 96 hospitals in various regions of India. This kind of analysis is the first of its kind in the country. However, the study has several limitations such as (i) HBCRs primarily capture data from patients who seek care at tertiary cancer centers or cancer-directed treatment centers. As such, the cases diagnosed and treated at smaller hospitals might be under represented. This might lead to over representation of more advanced cases. (ii) Patients from nearby states or districts might have sought treatment in institutions located in other regions, potentially affecting the accuracy of region-wise comparisons and patterns, (iii) HBCRs are unevenly distributed. The southern region has a higher concentration of reporting centers, which might contributed to the disproportionately higher number of thyroid cancer cases reported from this region, i.e., 42% of hospitals are from the South, (iv) HBCRs do not represent population-based data, consequently, the findings reflected only the profile of treated cases and not the true regional disease burden (v) other than age, gender and religion, data on demographic variables such as marital status, educational status, income and so on, are not available. Despite these limitations, the study offers a broad overview of thyroid cancer patterns across major regions in India and served as a foundation for population-based and translational research.

## Conclusion

In conclusion, we observed considerable heterogeneity in demographic, clinicopathological, treatment and waiting time distribution from diagnosis to CDT for TC across the regions in India. This provided information on the necessity for identifying whether the predominance in the South was attributed to over-diagnosis or whether any specific risk factors are associated with the high disease burden. The region-wise variation in the CED and treatment pattern suggests implementing a guideline for managing the disease that may help improve national and local cancer care practices and policies.

## Conflicts of interest

Nil.

## Ethical approval

The analysis was approved 3/2/2017 by the Institutional Ethics Committee of ICMR-NCDIR vide approval number NCDIR/IEC/2017/5.

## Author contributions

Concept and design: Dr Aleyamma Mathew

Interpretation of data: Dr Aleyamma Mathew, Dr Anita Nath, Mr Sudarshan KL, Dr Thilagavathi Ramamoorthy

Statistical analysis: Dr Preethi Sara George, Dr Thilagavathi Ramamoorthy

Drafting of the manuscript: Dr Aleyamma Mathew, Dr Preethi Sara George, Dr Anita Nath

Critical revision of the paper for important intellectual content: Dr Sreekumar A, Dr Elizabeth Mathew Iype, Dr Anita Nath

Data availability statement*:* Data supporting the findings of this study are available from the corresponding author [AN] on request.

## Figures and Tables

**Table 1.** Region-wise thyroid cancer from HBCRs in India by intention-to-treat.

Region	Radical		Palliative		Symptomatic		Unknown*		Total	*p* value**
	*n*	%	*n*	%	*n*	%	*n*	%	*n*	
North	1,247	82.6	250	16.6	10	0.7	3	0.2	1510	<0.001
East	211	68.5	39	12.7	18	5.8	40	13.0	308	
West	1,362	89.4	160	10.5	0	0.0	1	0.1	1523	
South	5,177	92.1	415	7.4	6	0.1	21	0.4	5619	
Central	81	76.4	24	22.6	1	0.9	0	0.0	106	
North-east	321	74.5	110	25.5	0	0.0	0	0.0	431	
Total	8,399	88.4	998	10.5	35	0.4	65	0.7	9497	

* excluded unknown for statistical significance

** *p* value is based on radical versus others (Palliative and Symptomatic)

**Table 2.** Region-wise thyroid cancer from HBCRs in India by waiting-time distribution (diagnosis to commencement of cancer-directed treatment).

Waiting time	Central		East		North		North-east		South		West	
	#	%	#	%	#	%	#	%	#	%	#	%
< 7 days	47	34.3	75	18.6	661	36.2	95	19.0	1,652	27.4	64	4.0
8–30 days	41	29.9	122	30.2	598	32.7	173	34.5	1,870	31.0	424	26.8
2–3 months	32	23.4	131	32.4	387	21.2	180	35.9	1,971	32.7	836	52.7
> 3 months	17	12.4	76	18.8	181	9.9	53	10.6	536	8.9	261	16.5
Total	137	100	404	100	1827	100	501	100	6,029	100	1,585	100

## Supplementary information

**Supplementary Table 1.** Region-wise thyroid cancer from HBCRs in India: female‑to‑male ratio by age.

Region	All ages		<35 years		35-49 years		50-64 years		>64 years	
	#	F:M	#	F:M	#	F:M	#	F:M	#	F:M
South	6,045	2.5	1712	3.5	2116	2.8	1531	2.0	686	1.5
North	1,827	1.6	508	1.8	546	1.8	514	1.5	259	1.3
West	1,607	1.7	562	2.1	541	1.8	371	1.4	133	1.3
Central	137	2.0	29	3.8	36	2.6	46	1.6	26	1.4
East	404	2.3	145	3.3	133	2.9	92	1.4	34	1.1
North-east	501	2.3	121	4.3	163	3.1	142	1.6	75	1.1

**Supplementary Table 2.** Thyroid cancer by histology: female(F)‑to‑male(M) ratio by age, histology and CED.

Histological classification			All cases	PC	FC	MC	AC	SCC	Ca. NOS	Others
Female‑ to‑male ratio by Age	All ages	***n***	10,521	8,374	605	466	268	193	362	182
		F:M	2.1	2.3	2.6	1.0	1.6	1.4	1.8	2.4
	< 35 years	#	3,077	2,762	119	100	5	24	37	22
		F:M	2.8	2.9	5.0	1.2	1.5	2.4	1.6	3.4
	35–49 years	#	3,535	2,943	185	183	44	47	71	42
		F:M	2.4	2.5	3.9	1.1	3.9	1.5	2.1	1.8
	50–64 years	#	2,696	1,921	208	141	108	78	145	70
		F:M	1.7	1.8	1.9	0.9	1.5	1.4	1.7	3.1
	> 64 years	#	1,212	748	93	42	111	44	109	48
		F:M	1.4	1.4	1.4	0.7	1.2	1.1	1.7	1.8
Histology versus CED	Localised	*n*	4,184	3,603	242	140	26	44	115	14
		%	40.3	43.3	41	30.1	9.8	22.9	31.9	7.8
	Loco-regional	*n*	4,678	3,815	164	240	130	116	143	70
		%	45.1	45.9	27.8	51.6	48.9	60.4	39.6	38.9
	Distant met.	*n*	1,474	867	184	84	110	32	101	96
		%	14.2	10.4	31.2	18.1	41.4	16.7	28	53.3
	Unknown	*n*	38	35	0	1	0	0	2	0
		%	0.4	0.4	0.0	0.2	0.0	0.0	0.6	0.0
	Total	*n*	10,374	8,320	590	465	266	192	361	180

*PC: Papillary carcinoma; FC: Follicular carcinoma; MC: Medullary carcinoma; AC: Anaplastic carcinoma; Ca. NOS: Carcinoma, not otherwise specified; SCC: Squamous cell carcinoma

## References

[1] Sung H, Ferlay J, Siegel RL (2021). Global Cancer Statistics 2020: GLOBOCAN estimates of incidence and mortality worldwide for 36 cancers in 185 countries. CA Cancer J Clin.

[2] Deng Y, Li H, Wang M (2020). Global burden of thyroid cancer from 1990 to 2017. JAMA Netw Open.

[3] ICMR-NCDIR (2020). Report of National Cancer Registry Programme.

[4] Pham DX, Nguyen HD, Phung AHT (2021). Trends in incidence and histological pattern of thyroid cancer in Ho Chi Minh City, Vietnam (1996–2015): a population-based study. BMC Cancer.

[5] Karkuzhali P, Yogambal M, Kumar M (2017). An Indian tertiary care hospital scenario of papillary carcinoma of thyroid. J Clin Diagn Res.

[6] Rao R, Giriyan SS, Rangappa PK (2017). Clinicopathological profile of papillary carcinoma of thyroid: a 10-year experience in a tertiary care institute in North Karnataka, India. Indian J Cancer.

[7] Das R, Sharma JD, Kataki AC (2019). Pattern of regional metastasis in papillary thyroid cancer: our experience of 86 cases. Int J Res Med Sci.

[8] Manjunath PR, Vadayath UM, Nair V (2020). Clinical profile of medullary thyroid carcinoma: audit from a tertiary care center in South India. Indian J Endocrinol Metab.

[9] Indian Council of Medical Research (2021). Clinicopathological Profile of Cancers in India: A Report of the Hospital Based Cancer Registries.

[10] WHO and ICD-10 (1992). International Statistical Classification of Diseases and Related Health Problems.

[11] Fritz A, Percy C, Jack A (2000). International Classification of Diseases for Oncology.

[12] Marascuilo LA, McSweeney M (1967). Nonparametric post hoc comparisons for trend. Psychol Bull.

[13] Mathew IE, Mathew A (2017). Rising thyroid cancer incidence in southern India: an epidemic of overdiagnosis?. J Endocr Soc.

[14] Veedu JS, Wang K, Lei F (2018). Trends of thyroid cancer in India. J Clin Oncol.

[15] Panato C, Vaccarella S, Dal Maso L (2020). Thyroid cancer incidence in India between 2006 and 2014 and impact of overdiagnosis. J Clin Endocrinol Metab.

[16] Mannathazhathu AS, George PS, Sudhakaran S (2019). Reproductive factors and thyroid cancer risk: meta-analysis. Head Neck.

[17] Donnay Candil S, Gorgojo Martínez JJ, Requejo Salinas H (2013). A retrospective cohort study of patients diagnosed of thyroid cancer in the southwest Madrid area. Predictive factors in differentiated thyroid cancer. Endocrinol Nutr.

[18] Linwa EMM, Ngom EM, Orock GEE (2021). Clinical profile and management of primary thyroid cancer in patients with nodular goitre at the Douala General Hospital, Cameroon. Pan Afr Med J.

[19] Li P, Ding Y, Liu M (2021). Sex disparities in thyroid cancer: a SEER population study. Gland Surg.

[20] Hoang JK, Branstetter BF, Gafton AR (2013). Imaging of thyroid carcinoma with CT and MRI: approaches to common scenarios. Cancer Imaging.

[21] Nguyen QT, Lee EJ, Huang MG (2015). Diagnosis and treatment of patients with thyroid cancer. Am Health Drug Ben.

[22] Lee JS, Leem JS, Yun HJ (2023). Aggressive subtypes of papillary thyroid carcinoma smaller than 1 cm. J Clin Endocrinol Metab.

[23] Eskander A, Devins GM, Freeman J (2013). Waiting for thyroid surgery: a study of psychological morbidity and determinants of health associated with long wait times for thyroid surgery. Laryngoscope.

